# Partitioning of red blood cell aggregates in bifurcating microscale flows

**DOI:** 10.1038/srep44563

**Published:** 2017-03-17

**Authors:** E. Kaliviotis, J. M. Sherwood, S. Balabani

**Affiliations:** 1 Dept. of Mechanical Engineering and Materials Science and Engineering, Cyprus University of Technology, Cyprus; 2 Dept. of Mechanical Engineering, University College London, UK; 3 Dept. of Bioengineering, Imperial College London, UK

## Abstract

Microvascular flows are often considered to be free of red blood cell aggregates, however, recent studies have demonstrated that aggregates are present throughout the microvasculature, affecting cell distribution and blood perfusion. This work reports on the spatial distribution of red blood cell aggregates in a T-shaped bifurcation on the scale of a large microvessel. Non-aggregating and aggregating human red blood cell suspensions were studied for a range of flow splits in the daughter branches of the bifurcation. Aggregate sizes were determined using image processing. The mean aggregate size was marginally increased in the daughter branches for a range of flow rates, mainly due to the lower shear conditions and the close cell and aggregate proximity therein. A counterintuitive decrease in the mean aggregate size was apparent in the lower flow rate branches. This was attributed to the existence of regions depleted by aggregates of certain sizes in the parent branch, and to the change in the exact flow split location in the T-junction with flow ratio. The findings of the present investigation may have significant implications for microvascular flows and may help explain why the effects of physiological RBC aggregation are not deleterious in terms of *in vivo* vascular resistance.

Red blood cell (RBC) aggregation is one of the key structural features of blood and a key determinant of blood viscosity. It is a physiological phenomenon, occurring mostly in athletic species, and is reversible in response to the shearing forces developed in the flow^1^,^2^. While it is generally accepted that in the healthy circulation the shearing forces are relatively high, minimising RBC aggregation, and ensuring low flow resistance, recent evidence has shown that aggregates persist throughout the microvasculature, implying that the red cell distribution and blood perfusion in the microvascular network may be affected by the intensity of the phenomenon^3^. Indeed, in a recent study examining the effect of RBC aggregation in blood flow through an artificial microvascular network a distinct increase in the capillary haematocrit was found^4^. The influence of aggregation on the passage of red blood cells through symmetric and asymmetric bifurcated vessels has been illustrated also in recent numerical studies^5^. Earlier studies on the RBC distribution in the microcirculation have clarified the factors, other than RBC aggregation, affecting the process; these were found to be the flow distribution in the bifurcations, the vessel geometry, the haematocrit in the feeding vessel, and the haematocrit profile in the vessels^6^,^7^.

Intense RBC aggregation and hyperviscosity syndrome are observed in many pathological conditions, altering the transport properties of blood. These conditions include sepsis^8^, sickle cell anaemia^9^, Alzheimer’s disease^10^ diabetes mellitus^11^ and rheumatological conditions^12^. However, the role of RBC aggregation and hyperviscosity syndrome, in vascular resistance remains unclear. For example, although hyperviscosity increases flow resistance, considered to cause local damages (e.g. by obstructing blood flow to the optic nerve in some instances of glaucoma^13^), it could also result in an increase of the luminal diameter of the vessels^14^ due to the production of nitric oxide. This would be in response to higher shear stresses acting on the endothelium and the consequence may be a reduction in flow resistance. Furthermore, *in vivo* experiments on the effect of RBC aggregation in whole organ perfusion show that there is a non-monotonic influence of the aggregation phenomenon on vascular resistance^15^. It is therefore essential to acquire a deeper understanding of the mechanical factors affecting blood flow and rheology in the microvasculature.

The key characteristics of microscale blood flows include the cell depleted layer (CDL) formed near the vessel walls, the spatial variation of RBC concentrations and the resulting plasma skimming phenomenon. The CDL is one way of describing the separation of the plasma and RBCs close to boundaries due to the radial migration of the cells towards the centre of the flow^16^,^17^,^18^. Recently, the effect of RBC aggregation on the CDL was studied in detail for flows in a T-junction microchannel by Sherwood *et al*.^19^ It was found that RBC aggregation a) independently enhances the width and roughness of the CDL and b) in combination with the flow partitioning in the bifurcation further exaggerates the width of the CDL. The spatial variation of RBC concentration in microscale blood flows is of particular interest since it gives rise to plasma skimming, and causes a reduction in haematocrit (the Fahraeus effect) and blood viscosity (the Fahraeus-Lindqvist effect)^2^. The haematocrit and spatial viscosity distributions in a T-junction microchannel were examined in a subsequent study by Sherwood *et al*.^20^ for both aggregating and non-aggregating samples and for a range of flow ratios Q*. The latter was defined as the portion of the flow rate between the daughter and parent branches (Q* = Q_D_/Q_P_). It was found that at low flow ratios RBC aggregation exaggerates the haematocrit reduction in the low flow daughter branch. The viscosity profiles, estimated using the empirical model of Pries *et al*.^21^, showed a strong haematocrit dependency in the daughter branches. The study was subsequently extended (Sherwood *et al*.^22^) to a sequential bifurcation, comprising T-junctions in square microchannels (50 × 50 microns in width and height) highlighting the influence of the local heterogeneous haematocrit distributions on the measured velocity profiles. The influence of RBC organisation in the parent branch, on the partitioning of the cells in bifurcations, has been shown in the recent study of Shen *et al*.^23^ A reversal of the well-known Zweifach-Fung effect at low haematocrits (<5%), with the lower flow branch receiving a higher haematocrit, was observed.

In all the aforementioned studies, information on structural characteristics, such as the aggregate size distribution in the flow was not examined. Such quantitative information on the intensity of aggregation at a local scale was provided by the authors^24^ for flows in a rectangular microchannel. A local aggregation index, A*, based on detecting the iso-intensity patterns formed by the aggregated cells was developed to characterise the aggregate size. This index allowed the quantification of the organisation of aggregates in the plane of shear and highlighted the combined effect of haematocrit and flow velocity on local aggregation characteristics.

Recently, Yeom *et al*.^25^,^26^ reported on the use of different aggregation measurement techniques to quantify RBC aggregation in a straight microchannel; methods used included ultrasound, erythrocyte sedimentation rate and speckle analysis in a rectangular geometry (1000 × 50 μm width and height channel). The speckle analysis was based on an intensity autocorrelation function, which provided a bulk index of aggregation. In the same study, the aggregate size distribution analysis was restricted to counting the number of RBCs per aggregate at low haematocrits (10%), in a very thin chamber (10 μm height), based on a method used in the rectangular microchannel study of Mehri *et al*.^27^ In the study of Mehri *et al*, aggregation of RBCs was quantified based on resolving aggregates in a rectangular geometry (110 × 60 μm width and height) and at low haematocrits (5, 10 and 15%) using image processing techniques. Aggregate size distributions were produced at different flow and pseudo-shear rates (from 2.5 to 11.0 s^−1^) and it was shown that the average aggregate size in the microchannel decreased with shear rate.

A review on aggregate characterisation using image analysis techniques is provided by Kaliviotis 2015^28^. In general image analysis for aggregate characterisation is more effective at low RBC concentrations and at narrow channel gaps, as in the abovementioned studies, for obvious reasons. However, such low haematocrits are non-physiological, and the interaction between RBCs and their aggregates is highly haematocrit dependent. A great number of additional techniques for aggregation characterisation at bulk levels exist in the literature, and commercial instruments are also available^29^. However, in microscale blood flows, and in particular in bifurcating geometries, where the flow and haematocrit conditions are not uniform, the local characteristics of aggregation are of particular significance. These characteristics affect the mechanical properties of blood, resulting in a local variation of viscosity (under the assumption of the fluid as a continuum). To the best of our knowledge, there are no studies in the literature quantifying local RBC aggregation characteristics in bifurcating microchannel flows.

In this paper, we report on a study of RBC aggregate size distribution in a T-junction microchannel for the first time, through analysis of the bifurcating flows previously reported by Sherwood *et al*.^19^,^20^ using the aggregate detection methodology developed by Kaliviotis *et al*.^24^

## Results

**Figure 1:**
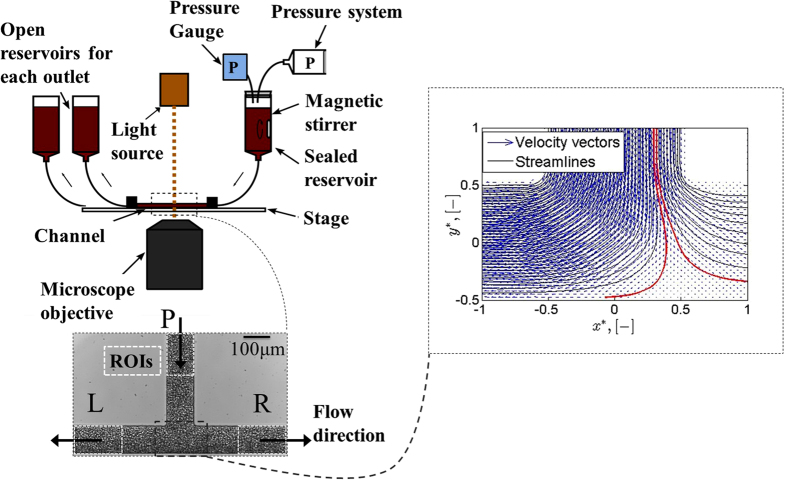
A schematic representation of the flow system. A pressure system was used to drive the flow entering the parent branch (P). The outlet left (L) and right (R) daughter branches led to the open reservoirs, the height of which was adjusted to provide the desired pressure drop, and thereby control the flow ratio. A magnetic stirrer was used in the inlet reservoir to avoid sedimentation and aggregation of cells in the samples. Regions of interest are shown as white, dashed rectangles. The velocity field and streamlines derived from an aggregating case with Q*~0.13 is shown in the right panel. The vector and streamline density is reduced by half for clarity of presentation. The red lines are the lines that separate the flow to the different daughter branches. The dimensionless quantities y* and y* are the x and y coordinates normalised by the channel width (100 μm).

**Figure 2:**
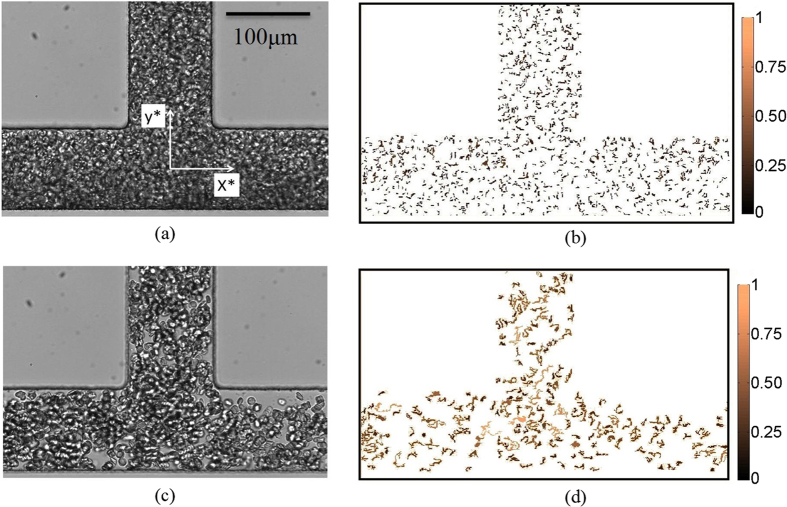
Representative cases of original and processed images. Top: (**a**) the original image and (**b**) the processed image of a non-aggregating sample (PBS) for Q*~0.5. Bottom: (**c**) original and (**d**) processed images of an aggregating sample (D2000) for Q*~0.2. The size of the detected structures is shown in colour-scale for a qualitative comparison; the size is normalised with the maximum size detected in the aggregating case. The origin of the global coordinate system is shown in panel (**a**).

**Figure 3:**
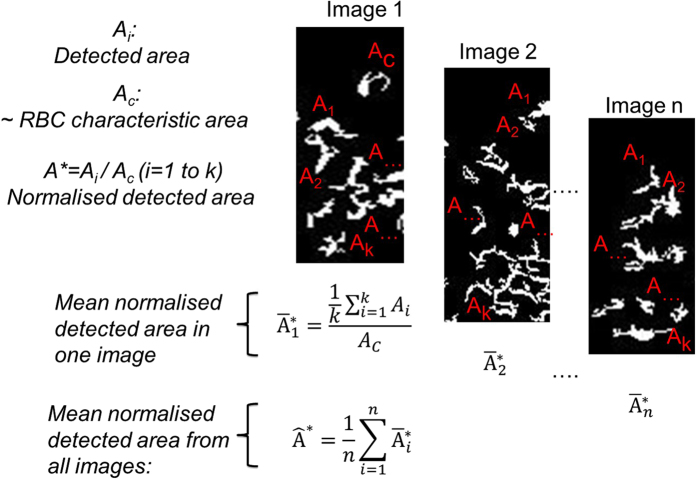
A schematic explanation of the key parameters quantified in the present study, A, Ac, A*, 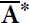 and 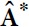. Images shown are magnified sections from representative processed images.

The experimental set-up shown in Fig. 1 was utilized in the study. The sample was perfused through a microchannel with dimensions of *W* = 100 *μ*m (width) and *D = *40 *μ*m (depth) by applying pressure to the sealed inlet reservoir. The distribution of the flow in the two daughter branches (flow split) was altered by changing the height difference between the two outlet reservoirs. The origin of the global coordinate system was placed at the intersection of the central axis of the parent and daughter branches and the coordinates were normalised with the channel width (x* = *x/W* and y* = *y/W)*. Figure 2 shows representative raw and processed images for the non-aggregating (PBS) and aggregating (D2000) blood cases, for flow ratios of Q* = Q_D_/Q_P_ = 0.5 and 0.2 respectively. The schematic in Fig. 3 illustrates the definition of the main quantities of interest in this work; the area of a detected structure in an image (A, in μm^2^), the normalized area of a detected structure (A*), the mean normalised area size in one image (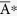), and the mean normalised area size from all images (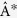). The normalising parameter is the characteristic area of one RBC (A_c_, in μm^2^). The size of aggregated structures present in the flow (i.e. the detected areas) was estimated using an edge-detection image processing technique^24^. The technique takes advantage of the fact that isointensity regions develop in the image as a result of the RBC aggregation process. Further details on the experimental parameters and methodology are provided in the *Methods* section.

### Aggregate size distribution and haematocrit

**Figure 4:**
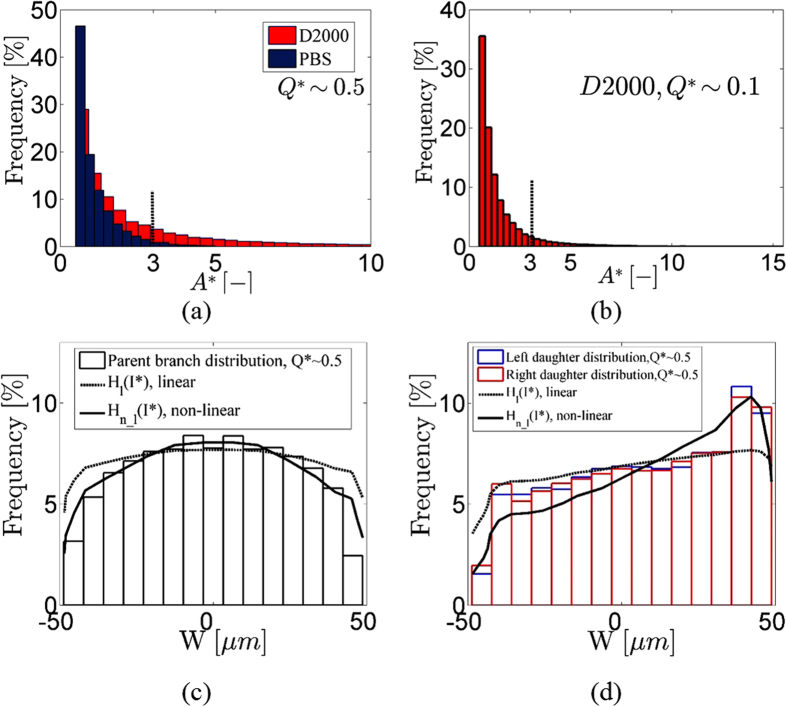
(**a**) Aggregate size (A*) distribution in the entire channel for the non-aggregating case (PBS) and for Q*~0.5 obtained from 400 images (*k* = 208479). The mean value is 1.06[0.50′, 1.08]. (**b**) detected edge area A* distribution for the aggregating case (D2000) obtained for Q*~0.5 and from 400 images (*k* = 73164). The mean value is 1.90[0.50′, 1.91]. The dotted line indicates the maximum area of one RBC. (**c**) distribution of all detected structures (A*) at Q*~0.5 in the parent and (**d**) daughter branches. Intensity-based haematocrit profiles (H(I*)) calculated as in Sherwood *et al*.^20^ (dashed line, linear function), and in Sherwood *et al*.^22^ (solid lines, non-linear function) are superimposed; in the present case the haematocrit profiles are scaled for the purpose of comparison.

Figure 4a compares the normalised size (A*) distributions of the structures detected in the entire channel (number of images *n = *400) for aggregating (D2000) and non-aggregating (PBS) cases respectively, and for a flow ratio of Q*~0.5. It should be noted that the distributions are left-truncated at 0.5 for the purpose of noise reduction. A* = 1 indicates the characteristic area of one RBC. However, red blood cells may adopt various configurations as they flow through the channel, altering the projected, and hence detected, RBC edge area in the images and resulting in the A* distribution shown in Fig. 4(a) for the non-aggregating case. The detected structures are significantly larger in the aggregating (D2000) case exhibiting a mean A* value of 1.90[0.50′, 1.91] (*k* = 73164, *n* = 400). Mean values are reported with their lower and upper bounds of the 95% confidence intervals, with the primed values indicating truncated data (*k* is the number of structures). A similar size distribution with a mean A* value of 1.88[0.50′, 1.90] can also be observed at a lower flow ratio (Q*~0.1) for the aggregating case (Fig. 4(b). These mean values suggest an almost twofold increase in the mean area size of aggregates compared to that of one RBC. It should be noted, however, that in the aggregating cases the area range of one RBC (minimum < A* < 3) does not necessarily imply structures of dispersed RBCs, since such areas could also be parts of aggregates. Excluding this area range from the distribution could provide information about larger aggregate characteristics; the mean area A* increases to ≈ 5.59[3′, 5.68] (*k* = 12378) in this case.

In order to examine the effect of local haematocrit on the distribution of A*, the spatial distribution of the detected structures across the parent and daughter branches is plotted in Fig. 4(c) and (d) respectively for the PBS case. These distributions show the location of all the detected structures in the channel irrespective of size. Corresponding haematocrit profiles estimated from the image intensity, H(I*), are also superimposed on the same figures. These intensity-dependent haematocrit profiles were developed in Sherwood *et al*.^20^,^22^ and represent linear (*H*_*l*_, dashed lines) and non-linear (*H*_*n*−*l*_, solid lines) relationships. In the parent branch (Fig. 4c) the distribution of detected structures generally matches the estimated haematocrit profiles. However, this is not the case in the daughter branches. This can be attributed partly to the assumed linear relationship between haematocrit and image intensity in the *H*_*l*_(I*) profiles, and to a small sensitivity of the image processing technique to the cell concentration (local haematocrit)^24^. Additionally, the calibration of the non-linear function *H*_*n*_*_*_*l*_(I*) was performed in a microchannel of slightly greater depth (50 μm cf 40 μm in the present study). It should be noted that no significant dependency of the spatial distributions on structure size was noted; i.e. spatial distributions plotted for different structure size ranges (for instance 1 < A* < 2, or 2 < A* < 3) remained qualitatively similar.

### Effect of flow ratio

**Figure 5:**
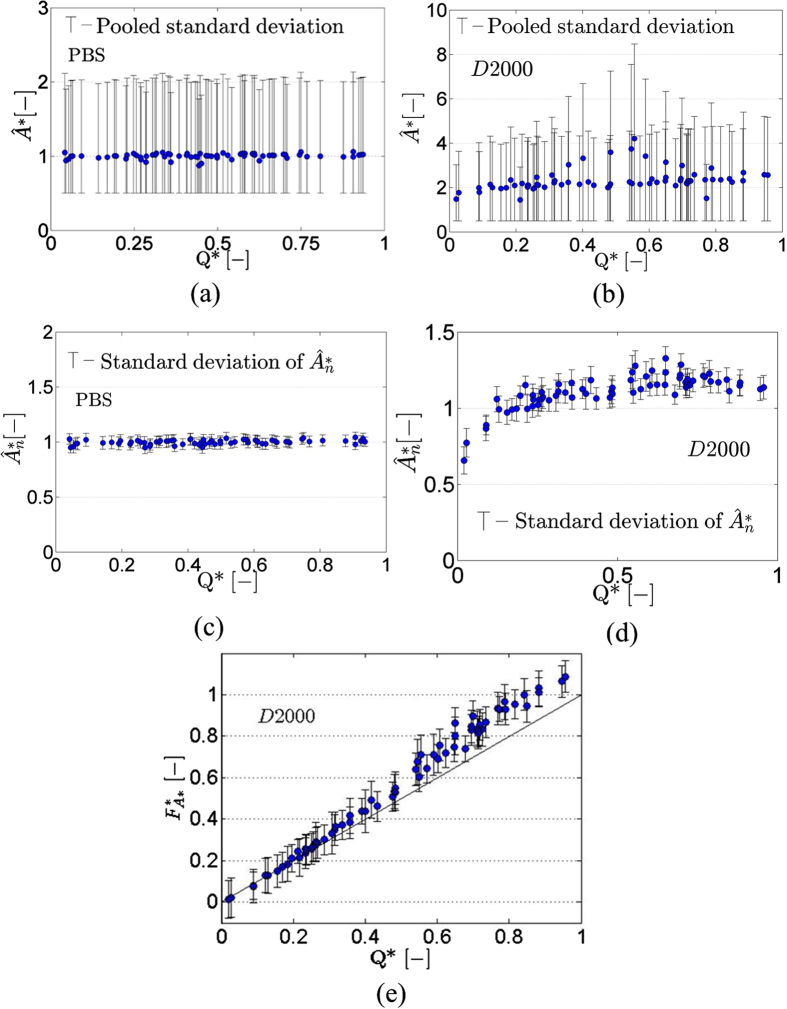
(**a**) Ensemble average (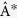) and 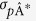 values based on 400 images against Q* for the PBS case. (**b**) Ensemble averaged values (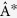) with pooled standard deviation for the D2000 case. (**c**) Ensemble averaged values 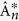 (PBS) obtained by normalising 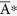 by the values in parent branch (the standard deviation of 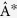 (n = 400) is also shown). The lower limit in the data of 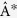 in (a) was 0.5 due to noise reduction in the data. (**d**) Ensemble averaged (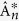) values of aggregate size 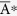 plotted against Q* (D2000 cases); the data is normalised by the 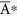 values in the parent branch. Data below Q* = 0.5 correspond to the right daughter branch and above Q* = 0.5 to the left daughter branch. The standard deviation of 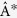 is also included. (**e**) Size-flow parameter F_A*_ = Q*A* against Q* as an index of the flow preference of different aggregate size.

Our previous work has shown that in the T-junction, the RBC concentration (haematocrit) decreases with the decrease of Q*^20^. For the non-aggregating case no dependency of the detected edge areas on flow ratio Q*, and therefore haematocrit, should be observed. This is illustrated in Fig. 5(a), by plotting the ensemble average 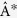 (n = 400) against Q*. The pooled standard deviation 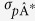 (equation 3 in *Methods* section) is shown in Fig. 5(a), and the standard deviation 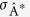 is shown in Fig. 5(c). The 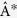 values in Fig. 5(c) were normalised by the values calculated in the parent branch and noted as 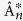. The magnitude of the pooled standard deviation seen in Fig. 5(a) relates to the fact that the random orientation the dispersed RBCs assume within the channel results in a range of detected structure areas. The low standard deviation values observed in Fig. 5c indicate the consistency of the processing and the reproducibility of the technique.

The flow ratio Q*, however, has a distinct effect on the distribution of aggregated structures in the channel. This becomes apparent when the relationship 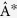 - Q* (Fig. 5(b) and (d)) is compared to that of the PBS cases. Figure 5(b) shows the ensemble averaged values 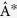 and the pooled standard deviation in the daughter branches as a function of Q*. The average size of the detected structures in this figure is approximately 2, with a tendency to decrease with decreasing Q*. The peak 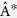 values observed around Q*~ 0.5 are due to increased aggregation in the particular sample; these data were not excluded from the following analysis, since the behaviour of 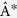 in the daughter branches is examined relative to 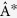 in the parent branches. Indeed, in Fig. 5(d) where the values of 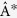 in the daughter branches are normalised with the 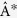 values calculated in the parent branch, no peaks are observed. Two important pieces of information can be extracted from Fig. 5(d): a) the mean aggregate size 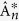 increases by ~10–20% compared to that in the parent branch for Q* values above ~0.2 and b) 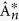 decreases for low values of Q*, namely below ~0.2. 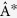 values tend to 1 at higher Q* ratios as expected. The preference of the larger aggregates for higher flow ratio branches is depicted more clearly in Fig. 5(e), where a size-flow parameter, F*_A*_ = Q*A*, is plotted against Q*; above approximately Q* = 0.3 the size-flow parameter F*_A*_deviates from the F*_A*_ = Q* line, indicating an increase of aggregate size relative to the mean aggregate size in the parent branch.

The decrease of 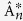, however, below Q* = 0.2 is counterintuitive considering the very low shear conditions in the daughter branches for these flow ratios, which should promote RBC aggregation. The cause of this behaviour can be traced to the structural characteristics of blood and the flow characteristics in the channel. As already mentioned, one of the main characteristics of the flow in the T-junction region is the flow split which is determined by the incoming flow (from the parent branch) and the pressure difference in the daughter branches. The flow split location in the parent branch, i.e. the boundary separating the flow entering each of the two daughter branches, is determined by the separating streamlines in the T-junction region (please see Fig. 1). The flow split location (denoted as 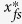) is expected to depend on the flow ratio Q* and hence to deviate from the channel centreline for asymmetric flow splits. 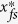 indicates the location of the flow split line on the x* axis.

### Partitioning of aggregates in the channel

**Figure 6:**
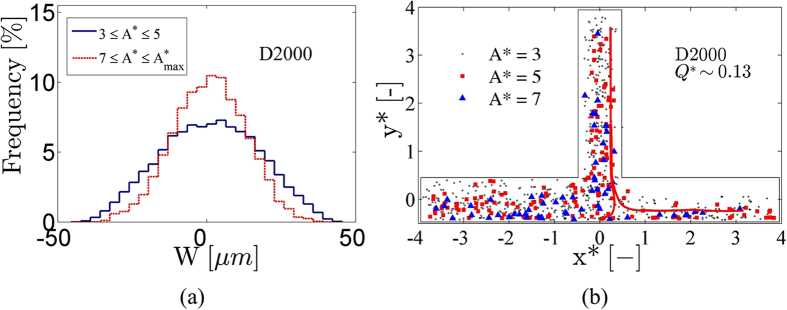
(**a**) Spatial distributions for two different A* ranges (3 < A* < 5 and 7 < A* < A*_max_) in the aggregating case (D2000, parent branch), and for all flow rates tested in this study (Q ~0.35 ± 0.5 μl/h). (**b**) Spatial distributions of RBC aggregates of three different sizes throughout the bifurcating microchannel geometry. A* ~7 is shown in blue triangles (▴), A*~ 5 is shown in red squares (▪) and A*~3 in black circles (⚬). The flow ratio in the left daughter branch is ~0.87 (Q* = 0.13 in the right daughter). The red flow streamlines define the flow separating lines in the parent branch. Selected aggregates are shown for clarity of presentation.

In order to examine the preferential location of the detected aggregates in the channel, spatial distributions for two distinct A* ranges (3 < A* < 5 and 7 < A* < A*_max_) were produced and shown in Fig. 6(a) for the parent branch and for all flow ratio cases examined in this study (for Q~0.35 ± 0.5 μl/h). The particular ranges of A* were chosen for illustrative purposes. Also, in order to exclude the sizes corresponding to one RBC, sizes up to A*~3 are excluded. The distributions in Fig. 6(a) illustrate that the larger structures (A*~7 to maximum size) tend to concentrate in the centre of the channel due to lower shearing forces therein, resulting in an aggregate depleted region near the wall. In addition, when larger aggregates form there is less cell-cell interaction and therefore the radial dispersion of the structures is decreased^30^; the wall-induced lift forces have a stronger effect in concentrating the aggregates towards the flow centreline. One implication of the concentration of the large aggregates around the flow centreline is a preference of these structures to flow towards the branch with the highest flow rate. This is apparent in Fig. 6(b) which shows the distribution of three different sizes of aggregates (A*~3 ± 0.02, 5 ± 0.02 and 7 ± 0.02) in the channel, for Q*~0.13. The flow separating streamlines are also shown in the figure to aid the discussion in the following sections.

### Effect of flow ratio on aggregate partitioning

**Figure 7:**
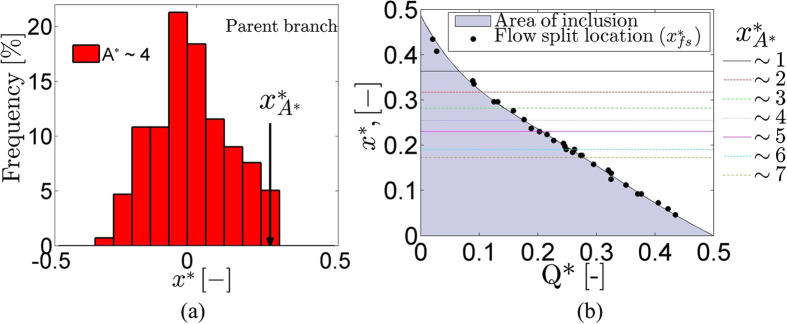
(**a**) Spatial distribution of a specific aggregate size (A*~4) in the parent branch; the vertical solid line represents the x* axis location of the mean value of A* calculated from the data falling below the 5^th^ percentile of the distribution (denoted as x*_A*_). (**b**) Location of the flow-split line on x* axis (x*_fs_) (black, filled circles) against Q*. The location of x*_A*_ is plotted in the same graph for specific A* values (1 to 7, coloured lines).

To illustrate the preferential concentration of larger aggregates towards the flow centreline in the parent branch and the resulting distinct aggregate depleted region near the wall indicated in Fig. 6, the spatial distribution of an arbitrary selected aggregate size (A*~4) in the parent branch, is plotted in Fig. 7(a). The arrow indicates the location of the mean value of A*, calculated from the data falling below the 5^th^ percentile of the distribution (right side); the location on the x* axis is denoted as 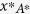. In the region between 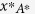 and the parent branch wall aggregates of size A* around 4 are generally absent. This region, together with the separating line for a given Q*, influences the partitioning of aggregates in the bifurcation and hence their distribution in the daughter branches. It is expected that for 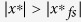, aggregates will flow into the adjacent daughter branch.

The flow-split location 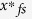 for all Q* values is shown in Fig. 7(b). The x*_A*_ locations in the parent branch, are plotted in the same graph as lines parallel to the x-axis, since they are independent of Q*. These are estimated from 400 images for each of the 7 distinct aggregate sizes (i.e. A* = 1 to 7) shown. The sizes of aggregates that will flow in a branch having a specific Q* value can be estimated from Fig. 7(b): for a specific Q*, the A* sizes found in the shaded area defined by the estimated low split locations are excluded from the particular branch, and only the A* sizes above a specific x*_f-s_ value (black-dots) will be transported to the relevant branch. For example, at Q*~0.2, aggregate sizes corresponding to values above the flow split location x*_fs_ for the given flow ratio, i.e. A* ranging from 1 to 5 are found. Aggregates in that size range will be transported into the branch with Q* = 0.2, whereas aggregates of all sizes will be transported to the opposite branch with Q*~0.8. Note that at Q*~0.5 the flow split line coincides with the centreline of the channel flow including all aggregate sizes.

## Discussion

The analysis of the non-aggregating cases (PBS samples) served primarily as a technique validation exercise. The main point of interest in these cases is the spatial concentration of structures in the channel, which has been studied previously^19^,^20^,^22^. Other interesting features of this flow, such as the orientation of RBCs and/or their frequency of rotation in the flow, could not be resolved at the haematocrit employed in the study. To study the details of the RBC motion at physiological haematocrit, either special treatment of the samples is needed^30^, or computational models can be employed^31^, although such models require rigorous validation with experimental data.

The analysis of the aggregating samples (D2000 data) revealed valuable new information regarding the partitioning of the aggregates in the T-shaped bifurcation and their distribution further downstream. In Fig. 5(d) it is observed that 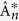 increases above 1 (i.e. exceeds the parent branch 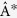 value) for Q* above ~0.2. This can be attributed to the reduction of the shear forces as a result of the lower flow rates in the daughter branches compared to the parent branch. Furthermore, after partitioning at the bifurcation aggregates are forced to one side of the channel causing a higher compactness and rate of interaction between them. Although the exact shear rate distribution in the channel cannot be obtained without knowledge of the flow field in the depth direction, it is reasonable to expect the mean shear in the daughter branches to scale with the mean velocity and hence with Q* (as the parent branch flow rate was approximately constant for all cases). An approximate measure of the level of shear is given by the ratio of the mean velocity (U), to the depth of channel, i.e. 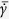 = U μm s^−1^/40 μm. The magnitude of this ‘pseudo-shear’ in the parent branch is approximately 8 s^−1^, which is considered moderate to low in terms of its influence on aggregate dispersion. In the daughter branches the pseudo-shear scales with Q* by definition, reaching very small values for the lower flow ratios.

The first novel piece of information extracted from Fig. 6(a) is that as the size of aggregates increases so does their distance from the side-walls of the channel. This behaviour is illustrated in Fig. 7b, where the distance x*_A*_ was plotted for various aggregate sizes (A* = 1 to 7). The development of aggregate structures of various sizes is mainly attributed to the flow conditions, and more specifically the local shear rates, which determine the magnitude of the shear forces in the channel. The dependency of the RBC aggregation phenomenon on shear rate is well established in the literature^32^,^33^,^34^. However, estimating the aggregate size distribution at physiological haematocrits is not a trivial task; hence it is not surprising that little such information exists in the literature. To the best of our knowledge no detailed analysis of aggregate characteristics in geometries more complex than a straight microchannel and at normal haematocrits has been conducted hitherto.

The behaviour of x*_A*_ in Fig. 7(b) implies that, in addition to the haematocrit variation in the cross-flow direction (illustrated in Fig. 4(c) and (d) and analysed in Sherwood *et al*.^19^,^20^,^22^), the size of the aggregates varies also. This implies that there are regions adjacent to the side-walls of the channel that are depleted of aggregates of certain sizes. Indeed, in Fig. 6(a) a clear region depleted of aggregates can be observed, although to term this an aggregate depleted layer would be an oversimplification, as the region is aggregate size-dependent. The implications of the existence of these regions can be seen in Figs 5(d) and 6(b), where a preferential distribution of aggregates is evident in branches with low flow ratio. Although the dependence of blood viscosity on RBC aggregation, in addition to other factors such as haematocrit, plasma viscosity, etc. is well established in simple geometries, the additional structural characteristics of aggregating blood found in this study provide significant information towards a better estimation of local viscosity profiles in more complex environments.

In a previous study investigating the local viscosity characteristics based on the spatial variation of RBC concentration we^20^ utilised the haematocrit-dependent viscosity model of Pries *et al*.^21^ to create viscosity profiles in the microchannel daughter branches for aggregating and non-aggregating cases, and illustrated the significant effect of RBC aggregation in this aspect. Local aggregation information, not included in the models of the aforementioned studies, was incorporated in another work by Kaliviotis *et al*.^35^, for aggregating blood flows within a parallel plate geometry. It was shown that significant spatial variation in the viscosity exists due to aggregate formation in the flow; the viscosity model used in that work was developed to account for various aspects of RBC aggregation including aggregate network formation^36^,^37^. The presence of the formed aggregates in the flow and the estimated distributions are expected to further affect the local viscosity of the fluid, since the mechanical characteristics of such structures are considerably different to those of RBCs in the dispersed state, even at similar local haematocrits. This is well established in the literature through studies such as the seminal work of Chien *et al*.^38^,^39^.

Accurate estimation of the local viscosity distribution in an aggregating sample, however, requires characterisation of the shearing conditions in the channel, which in the present analysis is restricted due to the lack of three dimensional velocity data, the aspect ratio of the channel and the depth of field of the microscopy system^24^. The RBC aggregates, however, could provide information on the extent of shearing they experience in the flow, as they are very sensitive to the local shear forces; in that sense, the aggregate size distributions presented in Fig. 4(a) and (b) imply that a range of shearing rates exists in the flow.

The aggregate size partitioning behaviour seen in Figs 5,6 and 7 may help explain why the effects of RBC aggregation are not as deleterious as expected^15^ in terms of *in vivo* vascular resistance; the smaller aggregates entering the low flow rate (and therefore low mean shear rate) branches, in combination with the lower haematocrit in these regions^20^, imply that the bulk viscosity in the branch will be kept low as well. This is a significant implication as it is known that blood exhibits yield stress^40^,^41^ at very low shear rates, which would have a negative impact if occurred in the low flow branches. Furthermore, since oxygen perfusion takes place mainly in the capillary level, the large CDL, and the low concentration of the aggregated cells in larger scale branches with low flow rates, as found in the present study, would serve the purpose of flow promotion. It should be noted here that the concentration of cells and aggregates in the low flow branch is strongly skewed towards the wall, i.e. in the lower velocity, but higher shear, regions of the flow. This may contribute to reducing the size of aggregates in the branch.

The present study considers a T-junction geometry (i.e. 90 degree symmetric bifurcations) which typically exists in the microcirculation^42^. To extent the findings of this study to other bifurcating geometries, such asymmetric T-junctions etc., the two main aspects that were found to affect the partitioning of aggregates should be considered: the flow split location x*_fs_ and the aggregate depleted region, defined by x*_A*_ in the parent branch. The aggregate depleted region is independent of the flow ratio and should depend on the aggregation intensity of blood. The flow split location, as shown in studies using bifurcations other than the T-junction^43^, seems to depend on the flow-split ratio in the outlets (Q*) only. This implies that the current findings could be extrapolated to geometries other than the T-junction. However, further experimental data might be required to verify this.

Another important flow parameter in the microcirculation is the haematocrit, as it largely affects the bulk and local viscosity of blood^20^,^21^. The haematocrit used in the present study (25%) is considered physiological for the length-scales studied^44^,^45^. However, the haematocrit may be altered in various pathologies. A change in the haematocrit - while flow conditions and RBC aggregation intensities remain the same - can affect the shear stress distribution in the flow, and subsequently the extent of aggregation, as the later depends on the shearing forces developed in the flow. The present study has shed light into important and previously unexplored structural phenomena of aggregating blood in the microcirculation. Nevertheless, further work is required to fully address both the influence of the bifurcation type and asymmetry, and the influence of haematocrit on local aggregation characteristics.

## Conclusions

RBC aggregation affects many aspects of blood flow at low shear conditions, including CDL, local haematocrit and local viscosity distributions, which have been demonstrated in previous studies by the authors^19^,^20^,^22^,^24^ using rectangular microchannels. In the present work additional structural characteristics, i.e. the size distribution of the formed aggregates in a T-shaped bifurcating flow, were quantified.

The significance of characterising aggregation at a local level is illustrated by the novel findings of this study; the analysis of the aggregate transport to the different daughter branches revealed a counter-intuitive behaviour between aggregate size and flow conditions, i.e. small aggregates found in branches of very low shear conditions in contrary to the current understanding on the aggregation/shear relationship. This phenomenon was attributed to the non-uniform distributions of aggregates in the parent branch, which in combination with the existence of a near wall region depleted of aggregates of certain sizes, affect the partitioning of aggregates for any given flow ratio, and hence the aggregate size distribution in the daughter branches. This new information on aggregate structure size and distribution could improve the estimation of local blood viscosity in the microscale and enhance further our understanding of microvascular flows. It may also help explain the contrast between *in vivo* and *in vitro* observations regarding the effects of RBC aggregation on vascular resistance or viscosity.

## Methods

The details of the experimental apparatus, sample preparation and flow measurement methodology can be found in Sherwood *et al*.^19^,^20^ whereas that of aggregate size characterization in Kaliviotis *et al*.^24^ Only a brief description is provided below.

### Sample preparation

Sample acquisition and all procedures involving human participation were performed with the approval, and in accordance with the relevant guidelines and regulations set by the Southeast London Ethics Committee (ref: 10/H0804/21). Blood was collected from healthy volunteers into vacuum tubes (BD) preloaded with 1.8 mg/ml EDTA after obtaining informed consent. The RBCs were washed twice in Phosphate Buffered Saline (PBS), centrifuged at 3000 rpm, and suspended in PBS containing D2000 (5 g/l). The haematocrit was adjusted to 25% by volume as appropriate for the microchannel dimensions used^44^,^45^. For consistency all experiments were conducted with a single sample.

### The flow system

A microchannel, with a width of *W* = 100 *μ*m and depth *D* = 40 *μ*m, was fabricated from SU8 using photolithography (Epigem, Redcar, UK), and is shown in Fig. 1. The sample was perfused through the microchannel by applying a pressure to the sealed inlet reservoir, relative to the open outlet reservoirs. The pressure in the inlet reservoir was controlled with an in house pressure control system using an actuated needle valve and a compressed nitrogen source. In order to minimise effects of RBC sedimentation, which is particularly pronounced for aggregating samples, the fluid in the inlet reservoir was continuously mixed with a magnetic stir bar, except when acquiring data, and all tube lengths were kept to a minimum.

For the cases studied, the inlet reservoir pressure was set to a single value, resulting at a mean velocity of 320 μm s^−1^ in the parent branch. The distribution of flow between the two daughter branches (flow split) was altered by means of hydrostatic pressure difference. The pressure difference was achieved by independently adjusting the height of the outlet reservoirs using micrometer stages. The flow split for a given acquisition case was then calculated using the PIV data, as described in the following paragraph. Between acquisitions, the channel was perfused at a high flow rate in order to ensure uniform hematocrit throughout the channel and system. Following, the pressure was reduced to the desired level and 20 s were allowed for aggregation to reach a steady state before acquisition proceeded^20^.

The origin of the global coordinate system is shown in Fig. 2a at the intersection of the parent and daughter branches. The coordinates are normalised with the channel width, x* = *x/W* and y* = *y/W*, respectively, and thus the parent branch width spans from x* = −0.5 to 0.5 and the daughter branches from y* = −0.5 to 0.5.

One T-junction channel was used throughout the experiment and 2000 images were acquired for each flow ratio Q*. The same blood sample was used for consistency at each Q* (in the range 0–1).

### Microscopy and microPIV system

The flow system was mounted on the stage of an inverted microscope (Leica DM ILM, Germany), with the focal plane set to the centre of the channel. A halogen light-source was used for illumination and images were acquired using an IDT X3 CMOS camera (Tallahassee, USA) at a frequency of 125 Hz. After pre-processing the images for alignment with the particle image velocimetry (PIV) interrogation windows, a final image size of 1216 × 700 pixels at a spatial resolution of 0.65 *μ*m/pixel was obtained. Multi-pass ensemble averaged PIV processing was carried out on each of the data sets, providing a final window size of 8 × 8 pixels and a vector spacing of 4 pixels (2.6 μm) using JPIV software (www.jpiv.vennemann-online.de/). A normalised median test was utilised to identify the invalid vectors, which were replaced with the median of the surrounding vectors. For the estimation of the flow rate for each branch, spatially averaged velocity profiles were acquired in the regions indicated by the region of interest in Fig. 1. The flow ratio was defined as the ratio between the flow rate in the daughter and the parent branch, Q* = Q_D_/Q_P,_ and was estimated from the average velocities (U) measured with PIV, according to Q* = U_D_/U_P_, as the cross-sectional area was the same in all branches.

The velocity fields were produced using the JPIV software. The velocity vectors are mean values from 2000 processed images. The data were further processed in Matlab to derive flow streamlines and in particular the separating streamlines that provide information on the flow partitioning at the bifurcation as a function of flow ratio (see Fig. 1). The velocity field and streamlines in Fig. 1 were derived from an aggregating case with Q*~0.13. The vector and streamline density is reduced for clarity of presentation. The red lines are the lines that separate the flow to the different daughter branches, and the point where these lines originate from in the parent branch is defined as the flow-split location in the x* direction (noted as x*_fs_). The flow split location was derived for all Q* cases analysed.

### Image based detection of aggregates

The method for detecting RBC aggregates in the acquired images was developed by taking advantage of the fact that connected RBCs (often termed rouleaux) result in continuous iso-intensity patterns, which can be detected with edge detection algorithms. In Kaliviotis *et al*.^24^ it was shown that these continuous areas of similar intensity increase almost linearly with the number of RBCs per aggregate, with relatively little deviation, for small aggregates of up to 15 RBCs per aggregate.

The detection algorithm is detailed in Kaliviotis *et al*.^24^ and briefly described below. It comprises the main image processing stages:

- *Image pre-processing to improve image quality*; these included: intensity transformations for improving the global contrast of the image^46^ and a correction of uneven illumination using a Gaussian function.^22^
- *Local intensity gradients (∇I*_*i*_*) identification*: the magnitude of the local intensity gradients was detected using an algorithm based on the Prewitt method^46^. Four intensity differences per distance (Δ*I*/Δ*S*) were computed from all eight opposite neighbouring pixels in a 3 × 3 pixel convolution window and then the magnitude of the local intensity gradient was calculated by applying spatial convolution:
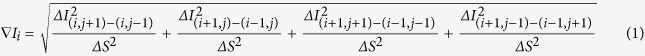
*ΔS* = 1.30 μm for the horizontal and vertical neighbouring points, and *ΔS* = 1.61 μm for the diagonal neighbouring points.The magnitude of the local gradient (∇*I*_*i*_) was normalised by the mean intensity of the convolved region (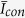): 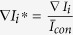, [*I* *μm*^*−1*^]. This was necessary because the gradients produced by aggregates are influenced by the background intensity; this dependency is complex to analyse when considering the whole gradient spectrum, however, when focussing only on the edges of RBCs it tends to be non-linear following a parabolic behaviour^24^.
- *Blurring correction*: small areas at the extreme ends of the daughter branches, were slightly blurred. Although this blurring does not have a significant effect on the cross-correlation process of the micro-PIV technique (as the PIV measurement is depth saturated), it does affect the local gradients 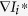 within those areas. Therefore a correction procedure was implemented for the blurred regions, which was based on creating a mean contrast image (n=2000) from the PBS case. This mean contrast image was created by calculating the local coefficient of variation of the pixel intensity values in the image 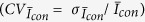 using a 3 × 3 convolution window, performed at each pixel. 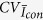 was normalised with its maximum value in the channel 
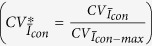
 producing an image with pixel values equal to 1, for the unaffected regions, and pixel values lower than 1 for the blurred regions (~0.87 was the minimum value). The affected gradients in the blurred regions were corrected using the following expression:
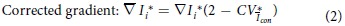
- *Edge detection*: The points in which the local gradients were above a certain threshold value were defined as edge points and converted to white (assigned a value of 1). Pixels with values below the threshold were assigned a value of 0. Calibration of the gradient threshold value was performed in the non-aggregating cases (PBS) and used blindly in the rest of the aggregation cases. It involved the identification of a threshold value that would result in a mean edge area (interconnected white pixels) equal to a characteristic RBC area A_*c*_. This characteristic RBC area was defined based on the fact that different configurations of one RBC could be seen in the images due to the rotation and deformation of the cells in the flow; the estimated edge size of one RBC when viewed from the flat side, i.e. its maximum edge size, was estimated at ~45 μm^2^, whereas the edge area of one RBC when seen laterally (the minimum value) was estimated to be ~10 μm^2^. A conservative value of A_*c*_ = 15 μm^2^ was set based on the fact that the various limitations imposed by the nature of the imaged data (dense suspension, overlapping of cells, relatively low resolution, etc.), affected the overall quality of the images and prevented, to a large extent, complete RBC edges (e.g. in their flat position) to be resolved.
- *Noise elimination*: The detected edges (defined by the interconnected white pixels) that were smaller than 20% of the mean RBC edge area were discarded as noise.
- *Statistical analysis*: Statistical information about the identified edges that appeared in the processed image was extracted for further analysis; this included number, area and location of aggregates.

The methodology has been validated against other image processing and electrorheology techniques^47^ in Kaliviotis *et al*.^24^ and its limitations have been discussed therein^24^. Figure 2 shows representative cases of processed images; the original image and the detected edge areas of a PBS case for Q*~0.50 is illustrated in 2(a) and 2(b), whereas the result for the aggregating case (D2000 sample) for a Q* ~0.2 is shown in Fig. 2(c) and (d). In 2(d) small areas (50% of the mean RBC edge area, ~8 μm^2^), have been omitted for clarity of presentation of the larger structures. The size of the structures in Fig. 2(b) and (d) is indicated by using a colour-scale for a qualitative comparison; the sizes have been normalised relative to the largest size in the D2000 case. As it can be seen in Fig. 2(b) there is a narrower size distribution (mostly dark coloured structures corresponding to smaller sizes) compared to the D2000 case in Fig. 2(d) (lighter colours corresponding to larger structures).

### Statistical analysis

The measured and calculated quantities (pixel intensity, velocity magnitude, edge area, etc.) are typically expressed in terms of spatially and/or temporally averaged values. The normality of the data distributions was assessed with the one-sample Anderson-Darling test, using Matlab (Mathworks) software.

For normal distributions, the mean values (MV) and standard deviation (SD) are presented as MV ± SD. For exponentially distributed data, which are observed for the spatially varying edge areas in one image, the mean values (MV) and their lower (LB) and upper bounds (UB) of the 95% confidence intervals were obtained using a least squares fitting function^48^. The notation used for the presentation of the aforementioned quantities is MV[LB, UB].

When truncated versions of known exponential distributions are analysed (left-truncated or left- and right-truncated), the confidence bounds are replaced by the truncated value. The truncated values are noted with a prime: MV[LB′ UB′]. Left-truncation is carried out for the purposes of noise elimination, as explained in the previous section, and left- and right-truncation occurs when a specific range of a distribution is analysed.

The detected edge area of a single structure in an instantaneous image is denoted by A and when normalised by the characteristic area of one RBC (A/A_c_) it is denoted by A* (see schematic in Fig. 3). The mean value of *k* detected structures in one image, normalised by the characteristic area of one RBC (A_c_), is denoted by 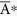 with standard deviation 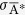. Note that for the exponential distributions of the spatially-varying A*, which is the case in the present work, 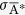 is equal to 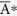 by definition.

When the time-varying mean value of 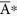 (denoted by 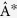) is calculated from a number (*n*) of images two relevant quantities regarding the dispersion of the data are relevant: a) the standard deviation of 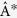, which is derived from the time-varying data and is denoted as 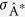 (note that 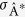 is calculated from normally distributed data), and b) the pooled (combined) standard deviation of 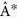, which is derived from the spatially-varying data in individual images and is denoted as 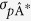; the latter is derived from exponentially distributed data, as explained above and provides a measure of the average dispersion of the data in the images. A sample of 400 images (*n* = *400*) was analysed (equispaced out of the 2000 images acquired) for all cases and the number of structures (*k*) is indicated in each different case. The abovementioned quantities are calculated from the variances:


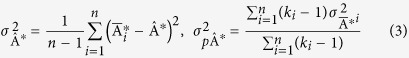


### Haematocrit Profiles

For comparison purposes haematocrit profiles derived in our previous studies were used. These haematocrit profiles were extracted from the data in Sherwood *et al*.^20^ (the same cases examined in the present study). It should be noted that for low haematocrits (~10%) the relationship between intensity and haematocrit could be assumed linear, however, for larger RBC concentrations a non-linear relationship exists^49^. Intensity-based haematocrit profiles are calculated as in Sherwood *et al*.^20^,^22^ for linear (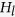, dashed lines) and non-linear (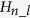, solid lines) haematocrit functions respectively:


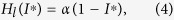



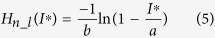


The constant α in *H*_*l*_ determines the actual haematocrit, however, in the present case it was adjusted for qualitative comparison purposes with the spatial distribution of A*; this was necessary due to the fact that the number of detected structures was not scaled for haematocrit. The values of the parameters *a* and *b* were defined in Sherwood *et al*.^20^,^22^ after calibration (*a* = 0.685, *b*=9.244) in a 50 × 50 μm rectangular microchannel.

### Limitations and error

The limitations of the image processing technique have been discussed in detail in Kaliviotis *et al*.^24^ The main factor affecting the image processing is the depth of focus which covers the entire depth of the channel, and therefore, overlapped cells appear in the image. This issue was addressed by calibrating the parameters for conservative processing, that is utilising the higher range of the image intensity gradients for the definition of edges and structures. This results in a relatively low density of detected structures, compared to the actual cells in the flow as already mentioned, and an underestimation of the aggregate sizes in the flow. Hence the main source of error in the data presented in this study is the parameter A*. Based on the validation process described in Kaliviotis *et al*.^24^ the error in the estimation of structure size A* might be of the order of 15% (estimated from the correlation curve of A* to actual aggregate size measured by inspection) with considerable deviation for aggregates consisting of more than 15 RBCs. Assuming that this error is systematic in the analysis it can be concluded that the trends shown in the present work are representative of the actual phenomena taking place in the flow configuration. Note that the standard deviation of the data in Fig. 5(d) is of similar order to the expected error.

## Additional Information

**How to cite this article:** Kaliviotis, E. *et al*. Partitioning of red blood cell aggregates in bifurcating microscale flows. *Sci. Rep.* **7**, 44563; doi: 10.1038/srep44563 (2017).

**Publisher's note:** Springer Nature remains neutral with regard to jurisdictional claims in published maps and institutional affiliations.
